# Structural and Functional Rich Club Organization of the Brain in Children and Adults

**DOI:** 10.1371/journal.pone.0088297

**Published:** 2014-02-05

**Authors:** David S. Grayson, Siddharth Ray, Samuel Carpenter, Swathi Iyer, Taciana G. Costa Dias, Corinne Stevens, Joel T. Nigg, Damien A. Fair

**Affiliations:** 1 Department of Behavioral Neuroscience, Oregon Health & Science University, Portland, Oregon, United States of America; 2 Center for Neuroscience, University of California – Davis, Davis, California, United States of America; 3 Institute of Psychiatry, Hospital das Clínicas, University of São Paulo Medical School, São Paulo, Brazil; 4 Department of Psychiatry, Oregon Health & Science University, Portland, Oregon, United States of America; 5 Advanced Imaging Research Center, Oregon Health & Science University, Portland, Oregon, United States of America; Beijing Normal University, China

## Abstract

Recent studies using Magnetic Resonance Imaging (MRI) have proposed that the brain’s white matter is organized as a rich club, whereby the most highly connected regions of the brain are also highly connected to each other. Here we use both functional and diffusion-weighted MRI in the human brain to investigate whether the rich club phenomena is present with functional connectivity, and how this organization relates to the structural phenomena. We also examine whether rich club regions serve to integrate information between distinct brain systems, and conclude with a brief investigation of the developmental trajectory of rich-club phenomena. In agreement with prior work, both adults and children showed robust structural rich club organization, comprising regions of the superior medial frontal/dACC, medial parietal/PCC, insula, and inferior temporal cortex. We also show that these regions were highly integrated across the brain’s major networks. Functional brain networks were found to have rich club phenomena in a similar spatial layout, but a high level of segregation between systems. While no significant differences between adults and children were found structurally, adults showed significantly greater functional rich club organization. This difference appeared to be driven by a specific set of connections between superior parietal, insula, and supramarginal cortex. In sum, this work highlights the existence of both a structural and functional rich club in adult and child populations with some functional changes over development. It also offers a potential target in examining atypical network organization in common developmental brain disorders, such as ADHD and Autism.

## Introduction

Human brain function is the result of a highly organized network of connections linking distinct areas across the brain. Recent work in neuroimaging has reflected a shift towards examining the brain in terms of its large-scale system dynamics [1], [2]. This shift could prove to be pivotal for clarifying the mechanisms that lead to both healthy and disordered brain function [3]. At the same time, identifying typical changes in the topology of brain networks across age will be crucial for generating an understanding of how complex human brain function arises.

One such topology hypothesized to exist in the brain is the so-called “rich club” organization, whereby the most highly connected nodes show a strong tendency to connect with other highly connected nodes. In recent years, rich club organization has been studied as an important indicator of certain functional features within many real-world networks. For instance, the protein-protein interaction network of the yeast *Saccharomyces Cerevisiae* [4] is absent a rich club, allowing for maximal functional specialization or biological segregation. On the other hand, rich club organization is a common feature of power grids and transportation systems [4], which likely allows for maximal integration and resilience to local disruptions.

Using Diffusion Tensor Imaging (DTI) and white matter tractography, van den Heuvel and Sporns [5] and van den Heuvel et al. [6] found a rich club of cortical brain regions in a cohort of healthy adults, consisting of medial parietal, medial frontal, and insular regions. The present study therefore asked: Does functional connectivity, as opposed to structural connectivity, show similar organizing principles?

Resting-state functional connectivity MRI (rs-fcMRI) examines the functional relatedness of brain regions based on correlated, spontaneous fluctuations of the blood oxygen level dependent (BOLD) signal while subjects are at rest [7], [8]. This method has been used extensively in the past to identify distinct functional systems such as the default mode network, the fronto-parietal/executive-control network, and the cingulo-opercular/salience network [9]–[11]. The integrity of these systems relate to subject performance across various cognitive systems [12] and has shown robust patterns of pathology within neuropsychiatric populations [13]–[15].

Importantly, although there appears to be a positive relationship between the strength of functional versus structural connectivity [16], [17], it remains unclear as to what extent the core topological properties exist across these two unique forms of connectivity and whether their developmental trajectories are unique or convergent. Developmental studies have found alterations in structural and functional connectivity as a function of age. Hagmann et al. [18] reported that local clustering of white-matter pathways decreased as a function of age and that structural modules became increasingly linked by long-distance pathways. However, the rank-order of node-centric measures (i.e. centrality) were largely stable. In a similar vein, Hwang et al [19] showed that the organization of functional hubs remains largely intact across development from childhood and into adulthood, although “spoke” connections – links from highly connected nodes to less connected nodes – appear to strengthen with age. These studies suggest that while overall hub organization may be relatively stable, age-related remodeling of brain networks does occur.

**Figure 1:**
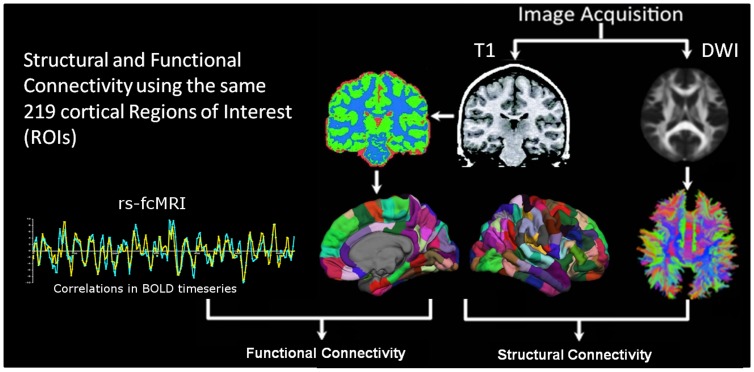
Overview of processing pipeline for each subject. *Region Selection:* T1-weighted image segmentation and parcellation resulted in a white matter mask for further diffusion data processing, as well as 219 cortical regions of interest (ROIs) covering the whole brain (the same ROIs were used for functional and structural analyses). *Structural Connections*: High-angular diffusion weighted MRI was acquired, and deterministic fiber tractography was performed throughout the white matter mask using a qball scheme. For each unique pair of ROIs, a connection weight was computed as the number of fibers with ends terminating upon them (see Materials and Methods). This resulted in a weighted network of structural connectivity across the whole brain. *Functional Connections:* Resting-state BOLD data (rs-fcMRI) was acquired, and timecourses were generated by averaging signal intensity across all voxels within a given region. Cross-correlations between regions were then used to generate the functional connectivity network.

In this report, we aimed to directly compare the whole-brain topology of structural and functional connectivity and assess their developmental trajectories. We used both high-angular resolution diffusion weighted imaging (DWI) and rs-fcMRI (see overview in Figure 1) to explore the structural and functional rich club organization in the brain in the same individuals. We then attempted to assess whether age-dependent structural or functional remodeling of rich club connections was observable by comparing a group of adults with a group of children. Given past work highlighting age-related strengthening of structural and functional connectivity, we hypothesized the rich club organization would be increased in adults relative to children.

## Materials and Methods

### Participants

A group of 14 healthy adults (aged 24–35, 4 male, 10 female) and 15 healthy children (aged 7–11, 8 male, 7 female) were included in this study. Experiments were approved by the Institutional Review Board at Oregon Health and Science University and conducted in accordance with the guidelines of the OHSU Research Integrity Office. For the adults participating in this study, written informed consent was obtained from each subject. For the children, written informed consent was obtained from the guardian of each subject, and assent from the child subject.

### MRI Acquisition and Processing

Imaging was performed during a single session for each participant on a 3T Siemens Tim Trio scanner with a 12-channel head coil. Data acquisition included a T1-weighted image for anatomical reference, a functional MRI scan, a T2-weighted image, and a high-angular resolution diffusion weighted image (HARDI). All participants completed all scans, except one child who did not undergo T2-weighted or diffusion-weighted scanning. An overview of the entire acquisition and processing pipeline is provided in Figure 1.

#### T1-weighed structural MRI and region selection

First, a whole-brain, high-resolution T1-weighted magnetization-prepared gradient-echo image (MP-RAGE) was acquired with the following parameters: repetition time (TR) = 2,300 ms, inversion time (TI) = 900 ms, echo time (TE) = 3.58 ms, flip angle (FA) = 10°, 1 mm^3^ voxels, 160 slices, FOV = 240×256 mm). Tissue segmentation into white and gray matter was performed on the T1 image using *Freesurfer* software (http://surfer.nmr.mgh.harvard.edu). Total brain volumes were quantified separately for children and adults and showed no significant group differences (1136+/−119 cm^∧^3 and 1215+/−144 cm^∧^3, respectively (p>.1)). Freesurfer was also used to parcellate the cortical gray matter into 68 regional labels in native space. In a second step, *Connectome Mapper* (http://www.connectomics.org/connectomemapper/) was used to further subdivide these regions into 219 cortical ROIs of roughly equivalent size and covering the entire brain (Figure 1; Table S1). Whereas previous studies on rich club phenomena used parcellations at either a very coarse resolution (82 regions) or a very dense resolution (1170 regions), here we examined rich club organization in a medium-density resolution of 219 cortical parcels. We considered this a useful strategy for validating past results, as prior work has demonstrated that network-based analyses of brain connectivity can vary substantially depending upon factors such as region selection and network size [20], [21]. In addition, these structurally based region sets have been used for functional connectivity analyses by other groups as well [18], [22]. Importantly, regions of interest were applied to both structural and functional data in each participant after surface registration, which is required for proper tractography and for assuring comparability of structural and functional data types. We note that while we chose this anatomically-based parcellation in part to maintain consistency with the initial reports of rich-clubness in structural brain networks [5], [6], other factors were considered as well. For example, biases can occur with tractography if region sizes are largely discrepant [1], and thus using parcellations based on functional networks (e.g. [10], [11] has its own limitations. Nonetheless, future work should consider functionally based parcellation sets as well.

#### Diffusion-weighted imaging

A HARDI scan was performed using an EPI sequence consisting of 72 gradient directions with b-value = 3,000 mm/s^2^ along with 10 unweighted B0 images. Acquisition parameters for the scan included the following: TR = 7100 ms, TE = 112 ms, 2.5 mm^3^ voxels, 48 slices, FOV = 230×230 mm. Diffusion data processing was carried out by *Connectome Mapper*, and consists of four stages: coregistration of the T1-weighted image and B0 images, diffusion data reconstruction, tractography, and identification of connections. We note that retrospective motion correction or motion censoring was not performed on DWI data due to lack of a currently established methodology, although future work should identify potential post-processing methods to attempt to correct for motion or alternatively to match samples based on motion parameters. In contrast, the rs-fcMRI data was scrubbed for excessive motion (see further below).

#### Coregistration of T1-weighted and B0 images

To facilitate accurate registration of the T1-weighted anatomical image onto the B0 image of the diffusion-weighted data, a T2-weighted image was acquired (TR = 3200 ms, TE = 497 ms; 1 mm^3^ voxels, 160 slices, FOV = 256×256 mm) as an intermediary. The following registrations were then carried out using FSL’s (http://fsl.fmrib.ox.ac.uk/fsl/fslwiki/) linear (flirt) and nonlinear (fnirt) registration tools. We performed a rigid-body transformation of the T1-weighted image onto the T2-weighted image, and then nonlinear registration of the T2-weighted image onto the B0 image, which allowed us to account for image distortion common in diffusion-weighted data, such as susceptibility artifact and eddy-current distortions. Every scan was then manually inspected to ensure high-quality accuracy for each step in the registration procedure.

#### Diffusion reconstruction

Diffusion data processing and tractography were carried out using the Diffusion Toolkit and TrackVis software (http://trackvis.org/blog/tag/diffusion-toolkit/) and consisted of the following steps. First, diffusion images were resampled into 2 mm^3^ voxel size and reconstructed using a Q-BALL scheme [23] into an orientation distribution function (ODF) at each voxel. The ODF was defined on a tessellated sphere of 181 vertices, and represents the estimated diffusion intensity in each direction. At each voxel, we defined up to 3 directions of maximum diffusion as defined by the local maxima of the ODF. This step is analogous to computing the principal eigenvector when using DTI.

#### Tractography

At each voxel of white matter, we initiated 32 evenly-spaced fibers for every direction of maximum diffusion. Each fiber was propagated in opposite directions, and upon reaching a new voxel, continued in the direction of whichever maximal diffusion direction was closest to its current direction. The growth process of a fiber was stopped whenever this resulted in a change of direction sharper than 60°, or when its ends left the white matter mask. Additionally, fibers shorter than 20 mm in length were considered potentially spurious and were removed. This resulted in a large sample of reconstructed white-matter fibers across the whole brain. We chose this approach for its straightforwardness in determining connected vs. unconnected nodes (as opposed to probabilistic methods, where post-hoc thresholding must be used. Note: this distinction is necessary for analysis of rich club organization), and to remain consistent with methods outlined in previous work examining structural rich clubs.

#### Structural connectivity

Structural connections between cortical ROIs were identified by combining the results of the tractography with the cortical parcellation. For example, two ROIs i and j were said to be structurally connected if there existed a fiber with endpoints in i and in j. Only fibers in which both ends terminated in a cortical area were included for analysis. This included 50–60% all reconstructed fibers for each subject. Connections were weighted by the total number of fibers between two ROIs, resulting in a 219×219 connection matrix of all possible ROI pairs. Average fiber length was also computed for each connection identified.

#### Resting-state functional connectivity (rs-fcMRI) acquisition

Functional data was acquired using a gradient-echo echo-planar imaging (EPI) sequence with the following parameters: TR = 2500 ms, TE = 30 ms, FA = 90°, 3.8 mm^3^ voxels, 36 slices with interleaved acquisition, FOV = 240×240 mm). Subjects were instructed to remain still and passively fixate on a crosshair for 10–25 minutes. Average scan time was 17.2 (SD: 6.4) minutes. Since prior work has shown that connectivity matrices are stable after 5 minutes of scan acquisition [24], we acquired lengthy scans in order to maximize the reliability of correlation coefficient estimates. We also note that our main findings regarding differences in rich club organization were insensitive to large changes in total scan duration (Table S2).

#### Rs-fcMRI processing

The raw fMRI data underwent standard fMRI preprocessing including slice-time correction, debanding, motion-correction, registration onto the T1 image, and resampling into 3 mm^3^ voxel size. Several additional steps were also taken to prepare the data for connectivity analyses [25], including temporal bandpass filtering (0.009 Hz<f <0.08 Hz), spatial smoothing (6 mm full-width at half-maximum), and regression of nuisance signals. The latter includes the whole-brain signal, signals from ventricular matter and white matter, and the six parameters related to rigid-body motion correction. Nuisance signals were also bandpass-filtered prior to regression [26].

#### Motion censoring

Subjects underwent several rigorous steps to correct for head motion during scanning. First, frame-to-frame displacement (FD) was calculated for every time point. FD was calculated as a scalar quantity using a formula that sums the values for framewise displacement in the six rigid body parameters (***FD_i_ = |Δd_ix_|+|Δd_iy_|+|Δd_iz_|+|Δα_i_|+|Δβ_i_|+|Δγ_i_|***, where ***Δd_ix_ = d(i−1)_x_ −d_ix_***, and similarly for the other five rigid body parameters) [27]. At each time point, if the FD was greater than 0.2 mm, the frame was excluded from the subject’s time series, along with 1 preceding frame and the two following frames [27]. Furthermore, if any participant had greater than 50% of frames removed, that participant was excluded from all analysis related to functional connectivity. On the basis of these criteria, 6 children and 2 adults were excluded leaving a final sample size of 12 adults and 9 children for functional analyses. Of the remaining samples, average frame removal was greater in children (mean: 33.2%; SD: 11.1%) than for adults (mean: 14.3%; SD: 14.4%). Therefore, we performed additional analyses to test whether group differences in rich club coefficients are related to lower frame removal in adults (Table S2) by randomly removing frames at multiple extents in adults, recomputing rich club coefficients, and re-testing for significant differences. We note that the same differences reported in the main text (increased rich club coefficients in adults across a wide range of k) were robust to large random frame removal, even down to only 5 minutes of remaining scan time.

#### Functional connectivity

The 219 cortical ROIs were first mapped from surface-space into the native T1 volume space of each subject. Analysis of the functional time series of each ROI was then performed using the co-registered fMRI image. Time series were computed by averaging the signal intensity across all voxels within an ROI for each time point. Cross-correlations were computed between the time series of all ROI pairs, yielding a correlation value between −1 and 1 for each pair. The final result was a 219×219-size correlation matrix for each subject.

#### Removal of adjacent connections

Structural and functional matrices were filtered through a final step in which connections between neighboring ROIs (ROIs sharing a border between voxels) were excluded. Typically, albeit for differing reasons, structural and functional data are biased toward short-range connections. As outlined by previous work [10], functional connectivity data often shows this bias due to nonbiological reasons such as partial voluming, movement, and the spatial blurring typically applied in the pre-processing stream. At the same time, tractography algorithms are typically less likely to “drop” a short-range fiber than a long-range fiber. Thus, connections between neighboring ROIs were excluded from our final analyses. Additionally, community detection was performed on the structural networks after further excluding connections where the average fiber length was less than 40 mm. We note that this protocol for excluding short-range connections was not itself a contributor to our findings of rich club organization, as removal of this filter led to functional and structural rich club coefficients that were substantially greater (data not shown).

### Group Networks

#### Structural

For both adults and children, a group-averaged network was computed according to procedures similar to those outlined in [5], [6]. From the set of individual connection matrices (14 adults, 36% male; 14 children, 50% male), only connections that were present in at least 50% of the group were selected for averaging (note, no participants were dropped at this step; here we are excluding connections, not people). Next, the group-averaged matrix was computed by averaging only across the cell values of the individual subject matrices that were nonzero (again, all connections present in less than 50% of participants were set to zero). This yielded connectivity matrices for children and adults that were well-matched in terms of connection density (5.4% and 5.6% respectively).

#### Functional

Within each group (here, 12 adults, 33% male, and 9 children, 56% male, after excluding subjects for head motion as explained earlier), group-averaged networks were created by averaging individual correlation matrices together. To enable graph analyses relevant to this study, negative connections were ignored, and group networks were thresholded to include only the strongest (most positive) correlations. The results shown in this paper with regard to rich club curves reflect the network thresholded at a connection density equal to that of the group structural network for the adults (5.6%), to enable comparison between functional and structural organization. In addition, comparisons were performed at a range of connection densities between 4%–10%.

### Graph Analyses

#### Community detection

The resulting networks were analyzed with graph theoretical methods [2]. Functional and structural group matrices were examined for community structure using the community detection algorithm for undirected, weighted matrices adapted from Newman [28] and freely available through the Brain Connectivity Toolbox (http://www.brainconnectivity.net). The algorithm provides a subdivision of a given network into non-overlapping groups of nodes (communities) in a way that maximizes the number of within-group edges, and minimizes the number of between-group edges.

#### Rich club organization

The rich club phenomenon is said to occur when the most highly connected nodes show greater connectedness to each other than expected by chance. This was examined in terms of both unweighted and weighted matrices (weighted by correlation values for functional networks, and by number of fibers for structural). First, the “degree” of each node was computed as the number of links to other nodes in the network. A subgraph of the original matrix was then constructed for each degree k, from 1 to the maximum k value, in which only nodes with a degree of at least k were included. For unweighted matrices, the rich club coefficient Φ(k) was then calculated as the ratio of the number of connections between nodes within the k^th^ subgraph and the total number of possible connections between them. This is given formally by the equation [4], [29]:
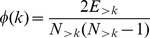



For weighted matrices, rich club organization was quantified along similar principles. Within each k^th^ subgraph, the number of all links E_>k_ were counted, and the collective weight of those links W_>k_ were summed. The weighed rich club coefficient Φ^w^(k) was then computed as the ratio between the sum of the subgraph weights W_>k_ and the sum of the strongest E_>k_ connections from the original weighted matrix. This is given formally by the equation [30]:
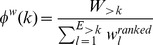



Next, we compare and normalize the rich club coefficient to sets of “equivalent” random networks. To do this, a thousand random networks were generated with equal size and degree distribution (or weight distribution for Φ^w^(k)). The rich club curve was computed for each random network, and then averaged across them to give Φ_random_(k). The normalized rich club coefficient Φ_norm_(k) was then computed for unweighted or weighted matrices, respectively, as:
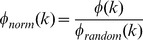


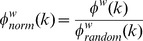



The network is said to have rich club organization when Φ_norm_ or Φ^w^_norm_ is greater than 1 for a continuous range of k [5].

In addition to normalizing networks with the classic Maslov-Sneppen rewiring, we performed separate normalizations using the Hirschberger-Qi-Steuer (H-Q-S) algorithm, which matches the transitivity that is inherent in correlation networks but does not preserve degree distribution [31]. Results for this testing are provided in the supplemental materials.

To assess statistical significance of the rich club curves, permutation testing was used [3], [32]. The set of 1000 random networks yielded a null distribution of rich club coefficients. Using this distribution, a p-value was assigned to Φ_norm_(k) as the percentage of random (null) values that exceeded Φ_random_(k) (*p<.05, one-tailed).

Differences in rich club organization between the adult group and the child group were also tested for significance using two different methods of permutation testing. In the first method, for each i^th^ iteration of the adult random network M_Ai_ and the child random network M_Ci_, the difference between the rich club coefficients for M_Ai_ and for M_Ci_ yielded a null distribution of 1000 random differences. Using this distribution, a p-value was assigned to each observed difference Φ_adult_(k)–Φ_child_(k) as the percentage of null differences that exceeded Φ_adult_(k)–Φ_child_(k) (*p<.05, two-tailed). Results using this method are provided in the main text. In the second method, group labels for children and adults were randomly reassigned to each subject. The rich club coefficient was then computed for each randomized group and the difference was computed and stored to build a null distribution. A thousand permutations were performed, and a p-value was assigned to each observed difference as the percentage of null differences that exceeded the observed difference. Results using this method are provided in the supplemental materials.

#### Community index (C)

In a previous report, van den Heuvel [5] used a measure termed “Participation Coefficient” to examine the level of participation of a node across communities and the level of community integration that node supports. This measure is scaled by 1) The number of modules the node connects to, 2) the number of connections (or distribution) for each module, and 3) the number of links to a node’s own community. In this report, we were primarily interested in identifying nodes with a high level of between-module connectivity, regardless of within-module degree. Therefore we introduced the following: Community index and Distribution index. These indices are independent of within-module connectivity, which distinguishes these measures from the participation coefficient. The Community index for a particular node is defined as the sum of the number of connections the node has with modules other than the module to which that node belongs. Thus, it gives us the direct level of community integration for that node without considering the number of connections to any given community (including its own). The community index is formally given by the following:
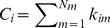



With N_m_, the number of modules excluding node-module (m_i_); and k_im_, the connection between the node and the module (is 1 when connected and 0 when not connected).

#### Distribution index (D)

The Distribution index for a node indicates how distributed its outside community links are. The Distribution index is calculated by the following steps:

Step 1: Compute the number of connections the node has with every outside community it connects to.

Step 2: Calculate the relative differences in outside community links. This is defined as the difference between the number of connections to two communities, for each unique pair of outside communities the node connects to. Compute the mean of relative differences (*d*_i_) for the node.

Step 3: Find the maximum value (*max(d)*) of mean relative differences across all nodes, and subtract each *d*_i_ from this max(*d*).

Step 4: Weight the subtracted value by multiplying it to the corresponding node’s community index (C_i_).

The D_i_ can thus be expressed by:
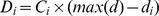



#### Adults vs. children comparison using the Network-based Statistic

The Network-Based Statistic (NBS) [33] is a recently established approach for identifying clusters of connections within a network that significantly differ between two sample populations. It has recently been applied to task-related functional [34], resting-state functional [35], and structural [36] connectivity networks. Here it is used to search for differences in the full, unthresholded functional connectivity matrices of adults and children. Its main advent lies in the way it controls for the family-wise error rate, which differs from more traditional, conservative approaches such as Bonferroni correction or the use of the false discovery rate, which assess the existence of an experimental effect at the level of each connection by correcting for the total number of multiple tests. By contrast, the NBS looks for an experimental effect at the cluster level, according to the following procedures (for in-depth documentation, see Zalesky et al. [33]).

First, a test statistic (T) is computed for every connection individually. Then, a threshold is chosen so that only connections exceeding a given test statistic are considered. Among these supra-threshold connections, the NBS searches for clusters. Two connections are considered clustered together when they share a common node, and the total number of connections constitutes the cluster size. Permutation testing using 10000 iterations is then performed to generate a null distribution of the largest cluster size. From this null distribution, a family-wise corrected p-value is assigned to each observed cluster.

## Results

### Similarities Exist between the Modular Organization of Structural and Functional Connectomes

We began our analysis by attempting to detect the community structure in both our structural and functional matrices (i.e. the connectomes) in adults. Community structure refers to the appearance of densely connected groups of nodes (i.e. brain regions), with only sparse connections between the groups. Previous work across the connectome using functional data has consistently identified multiple distinct communities of regions consisting of both sensory-related and control-related systems [10], [11], [37], while with structural data the results have been mixed [5], [18].

**Figure 2:**
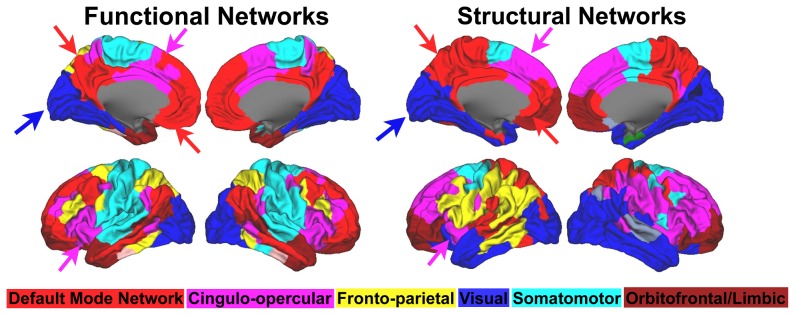
Community detection in functional and structural group networks from healthy adults. Brain regions are colored according to which community they belong to. In the functional network (left), six predominant communities were identified, comprising the Default Mode (red), Cingulo-opercular (pink), Fronto-parietal (yellow), Visual (blue), Orbitofrontal/Limbic (dark red), and Somatosensory (light blue) systems. Communities resembling analogues of the functional systems were identified in the structural network as well (right).

With regard to the functional data (Figure 2), and in agreement with this prior work, we identified six prominent functional communities in adults. These communities consisted of the default mode, cingulo-opercular, fronto-parietal, visual, orbitofrontal/limbic, and somatomotor systems.

For the structural matrices (Figure 2) we also found large-scale communities that, while not identical, share largely overlapping region sets. For instance, the grouping of bilateral PCC/precuneus together with the ventral medial prefrontal cortex/rostral cingulate forms a plausible analogue of the default mode network (red arrows in figure), while the grouping of dorsal ACC with the anterior insula looks like a unique version of the cingulo-opercular system (maroon arrows in figure). These findings are consistent with the idea that the structural and functional connectome share some core common features. While many studies have related the strength of functional connectivity to the strength of structural connectivity (see discussion), these data do show similarities in whole-brain organization as well.

### Structural Connectivity shows a Robust Rich Club Distributed across Several Brain Systems/Networks

**Figure 3:**
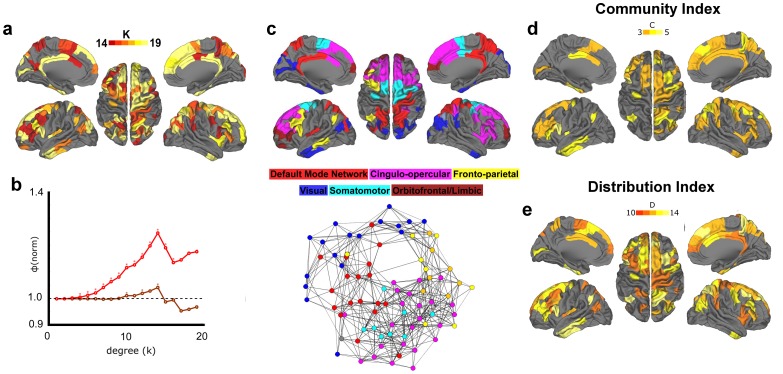
Rich club phenomena in structural group network of adults. (**a**) Regions comprising the structural rich club are displayed on an average brain surface. Degree k> = 14 was used to define rich club nodes, reflecting the peak value observed in the weighted rich club coefficient curve in (b). Results highlight the involvement of medial parietal/PCC, superior frontal/ACC, insula, and inferior temporal cortex. (**b**) Rich club coefficients relative to random are shown as weighted in red and as unweighted in dark red. Significant values (p<.05) are signified with an asterisk. (**c**) Rich club regions from (**a**) are colored according to community assignments. Below, a spring embedded graph shows rich club nodes and links between them, reflecting a high level of integration between systems. (**d, e**) Rich club regions with a high Community Index (C > = 3) and a high Distribution Index (D> = 10) are colored. A large proportion of regions are colored, reflecting high levels of integration.

We next attempted to identify the existence of rich club organization using the structural matrices of the adult participants. In agreement with past results [5], [6], we found significant rich club organization in the group structural connectome across several levels of k (i.e. degree). Figure 3b summarizes our statistical findings for both weighted (by number of fibers) and unweighted graphs.

The regions comprising the rich club are distributed bilaterally and include anterior and posterior cingulate cortex, superior frontal, superior parietal, and insula cortex, as well as the inferior temporal and fusiform cortex (Figure 3a). While the latter findings are unique to this sample, the overall patterns are largely consistent with prior reports [5], [6]. Importantly, here we see that the rich club includes a subset of regions from all the major communities identified in the structural connectome (Figure 3c). As visualized with the spring embedding diagram of the rich club nodes, these data may highlight at least one route on which data may be integrated between otherwise segregated large-scale brain systems.

To quantify some of these qualitative phenomena, we generated two indices aimed to calculate the extent to which these nodes connect to communities (systems) outside of their own (see Materials and Methods). The first, the community index (C), quantifies the number of communities a given node links to outside of its own. The second, the distribution index (D), quantifies the number of communities a given node connects to and considers the overall distribution of those connections. For example, if Node A connected to three communities but the distribution of these connections was skewed heavily towards one community, it would have a lower distribution index than node B who has an equal number of connections to all three. As shown in Figures 3d and 3e, these indices indicated that the nodes of the rich club generally connect to multiple communities, and that links to the multiple communities are generally equally distributed.

### Functional Connectivity shows Rich Club Organization as well

**Figure 4:**
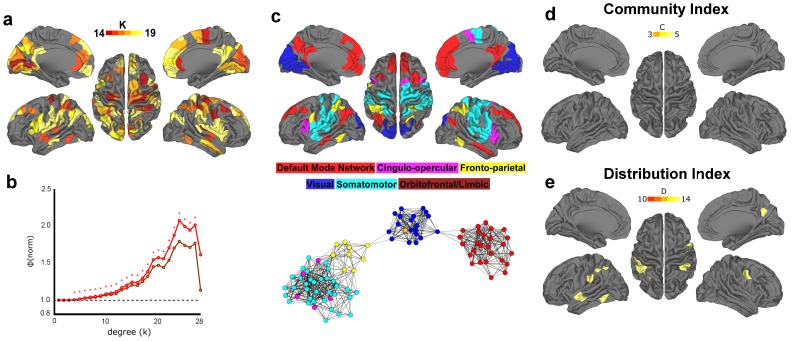
Rich club phenomena in functional group network of adults. (**a**) Functional rich club regions were defined as having degree k> = 14, equal to the degree threshold for the structural rich club. In agreement with prior research, these results highlight the involvement of medial parietal/PCC, medial frontal/ACC, and insula cortex. (**b**) Rich club coefficients relative to random are shown as weighted in red and as unweighted in dark red (*where both curves are significant, p<.05). (**c**) Rich club regions are colored according to which community they belong to. Below, spring embedded graph of rich club nodes and links between them, reflecting a low level of integration between systems. (**d, e**) Rich club regions with a high Community Index (C > = 3) and regions with a high Distribution Index (D> = 10) are colored. Nearly all regions are subthreshold, indicating very low levels of integration.

We next turned our focus toward the functional connectome. Figure 4b shows the normalized rich club coefficients of the adult functional matrix. For both weighted and unweighted matrices, rich club organization of the functional connectome is quite high and significant across nearly all k levels, excluding just the upper and lower ends. Rich club organization was additionally confirmed for a wide range of k using the H-Q-S normalization (figure S1).

**Figure 5:**
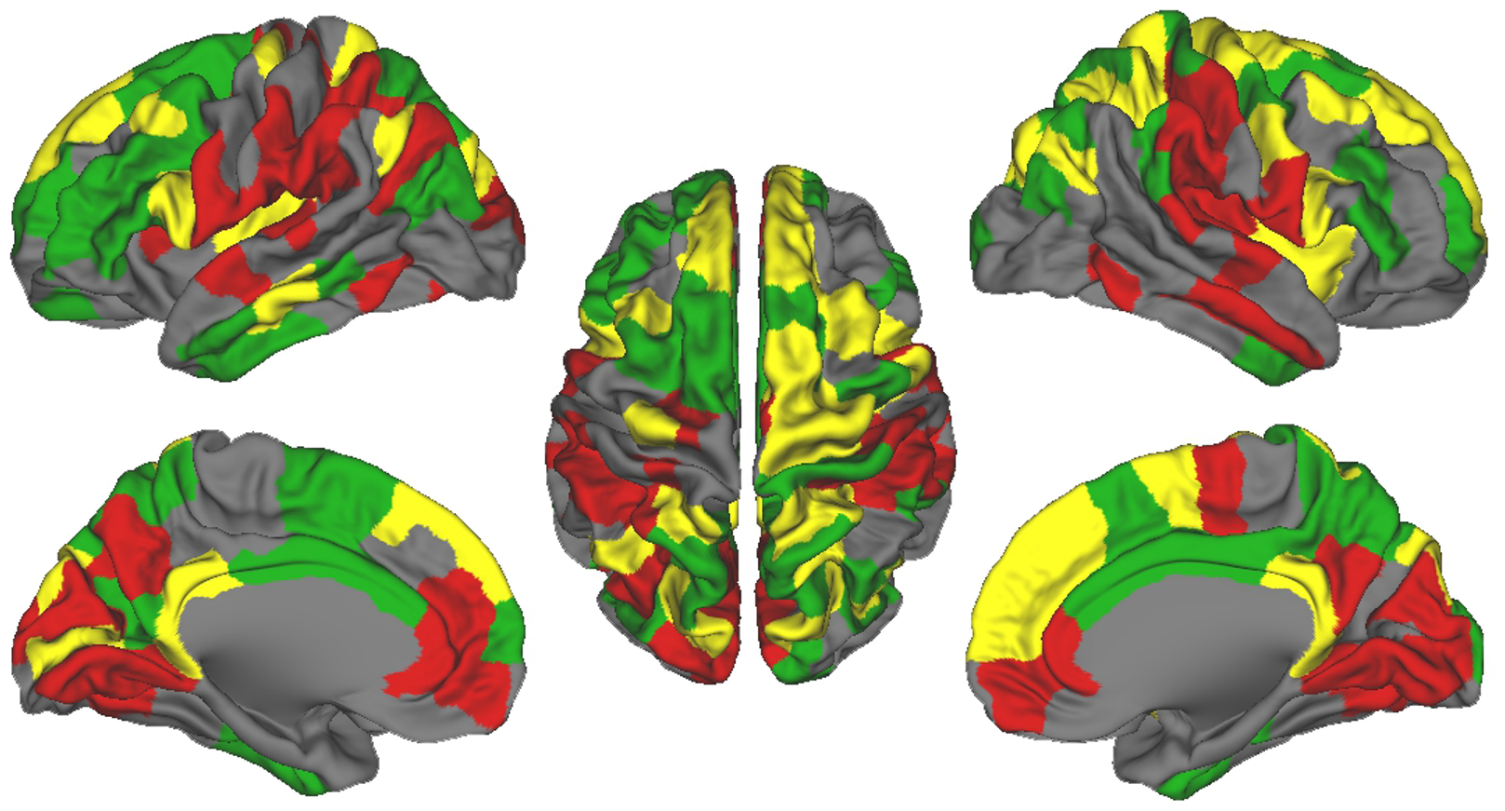
Regions that overlap between functional and structural rich clubs in adults. Overlapping regions, colored in yellow, defined as having degree k> = 14 in both the structural and functional group networks (the rich clubs). Structural-only and functional-only rich nodes are colred in green and red, respectively.

Much like the structural rich club, the regions in the functional rich club are also distributed preferentially along midline anterior, midline posterior, and insula cortex (Figure 4a). This phenomenon, capturing the similarities across the rich clubs, is visualized in Figure 5, showing nodes present in both the functional and structural rich clubs. We found that 34 nodes overlapped among a total of 92 structural rich nodes (37%) and 81 functional rich nodes (42%). Another correspondence between the structural and functional matrices regards the systems represented. The functional rich club nodes are dispersed broadly among the default mode, cingulo-opercular, visual, somatomotor, and fronto-parietal systems (Figure 4c).

Although there were striking and important similarities between the nodes that comprise the rich club for both functional and structural data, marked discrepancies were also observed. As visualized with the spring embedding diagram of the rich club nodes (Figure 4c), we can see that, unlike the structural data, most rich club nodes in the functional data are strongly and primarily connected within their own system. While there are some exceptions, notably connections between the somatomotor and cingulo-opercular systems, few connections are identified among the rich club nodes that integrate across systems. This phenomenon is captured in our community (C) and distribution (D) indices, which show that few nodes had strengths equal to that of the structural rich club (Figures 4d and 4e).

### Children have Highly Similar Structural Rich Club

**Figure 6:**
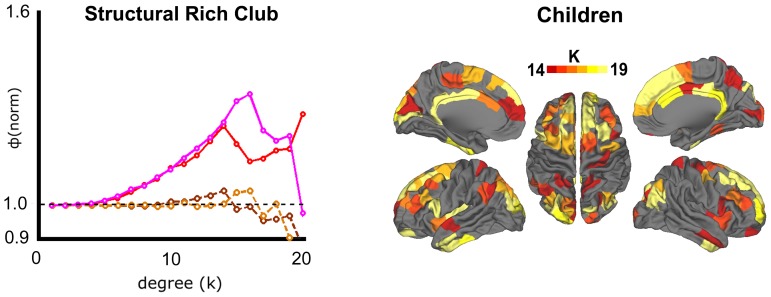
Comparison of structural rich club organization in adults versus in children. (Left) Normalized rich club coefficients for structural data are shown for weighted (adults = red, solid; children = pink, solid), and unweighted (adults = brown, dashed; children = tan, dashed) networks. No significant differences were observed for weighted or unweighted coefficients. (Right) Regions with degree k> = 14 are shown, to facilitate direct comparison with adults. Results indicate substantial overlap in spatial layout with adults (Fig. 1a).

We concluded our analyses with a brief examination of rich club organization in a population of children (n = 14). Using the exact same procedures of analysis, and starting with the structural matrices, we identified a rich club organization that was highly similar to the adult population (Figure 6, right). Among the 81 adult rich nodes and 70 child rich nodes, 57 nodes (70% in adults; 81% in children) overlapped. Of the remaining 24 nodes in adults, 21 were adjacent to (sharing a border with) child nodes. Likewise, 12 of the remaining 13 nodes in children were adjacent to adult nodes. Regarding rich club coefficients, both populations showed a continuous range of values that significantly deviates from random and peaks near a k level of 15 (Figure 6, left). We tested a comparison of the rich club coefficients in the children and found no significant differences at any k level, and only at one point using the group-randomization method (figure S2). The regions comprising their rich clubs are also highly overlapping, forming the same profile of midline frontal, midline posterior, insula, inferior temporal, and cingulate cortex. Taken together, these findings support the notion that the structural rich club is already well-defined by late childhood (age 7–11).

### Difference between Age Groups are Observed in the Functional Rich Club

**Figure 7:**
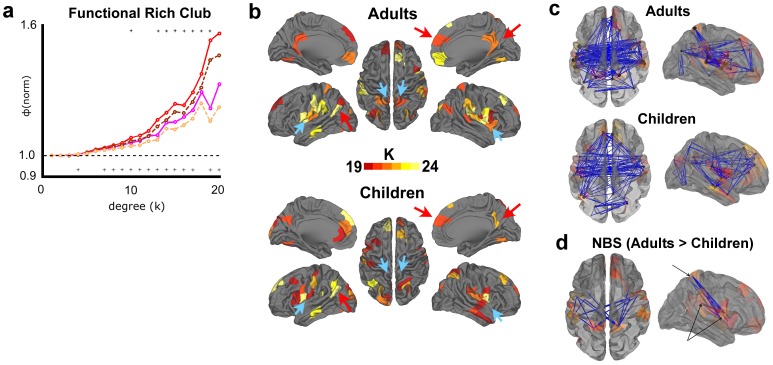
Comparison of functional rich club phenomena and network organization in adults versus in children. (**a**) Normalized rich club coefficients for functional data are shown for weighted (adults = red, solid; children = pink, solid), and unweighted (adults = brown, dashed; children = tan, dashed) networks. Significant differences are indicated with an asterisk at the top of the graph for weighted networks, and at the bottom for unweighted. Significant differences are observed (adults>children) across a wide range of k. (**b**) Rich club regions are displayed for the point of maximal difference in rich club coefficients that was significant (k> = 19). While many regions overlap (red arrows, for example), there are bilateral regions that appear only in adults (blue arrows, for example). (**c**) Rich club connections (k> = 19) are depicted for adults and for children. (**d**) Comparisons of the full, unfiltered matrices for adult vs. children subjects (non-group-averaged) using the Network-Based Statistic shows a single bilateral cluster of connections between regions of the insula, supramarginal, and superior parietal cortex. Cluster size was significantly greater in number than what we would expect at random (p<.01, one-tailed, T>4.5; significant clusters centered around the same nodes were also observed at T>4 and T>5 thresholds (p<.05, data not shown)). These connections primarily linked regions of the adult functional rich club as seen in (b) (k > = 19; lightly colored).

With regard to the functional connectome, we found an increase in the rich club coefficient across a broad and consistent range of k levels. Furthermore, this increase was greater than what we would expect to see between random networks, for k levels between 7 and 21 (Figure 7). Comparisons using group randomizations yielded weaker, but largely consistent findings (figure S2). This comparison was performed using connection densities equal to the density of the adult structural matrix (∼5.6%), although comparisons of group functional data performed at 4, 6, 8, and 10% densities yielded consistently significant results as well (figure S3). In addition, comparison of rich club curves using H-Q-S normalization confirmed increased rich-club organization in adults (figure S1).

To compare the organization of adult and child rich clubs, we mapped the regions onto the surface for both children and adults at k> = 19, which represented the peak difference in rich club coefficients that was significant (Figure 7a and 7b). The pictures show us a high level of similarity, including medial prefrontal, PCC/precuneus, inferior parietal, ventral visual, ventral somatomotor, and several regions in and around the insula (Figure 7b, red arrows), suggesting similar overall rich club patterns despite the decrease in strength in children. Each group had exactly 36 rich nodes, 20 of which (56%) overlapped. Of the remaining 16 nodes in the adults, 12 were adjacent to one or more rich nodes in the children. Likewise, 11 of the 16 rich nodes in the children were adjacent to adult rich nodes. With that said, there are at least 3 bilateral groups of nodes present in the adult group that are not present in children. These nodes include the dorsal aspect of the superior parietal cortex, the supramarginal cortex, and a large portion of the insula (Figure 7b, blue arrows). Group comparisons of the full matrices of individual subjects using the Network-Based Statistic [33], [35] show a cluster of connections that are greater in adults that seem to be most influential in these rich club changes (Figure 7d). These connections adjoin regions of the dorsal superior parietal, supramarginal, and insula cortex, and are indeed the same regions identified as being integrative across communities within the adult rich club and as being absent from the child rich club.

## Discussion

Neuroimaging studies have increasingly relied on graph theoretical methods to explore questions about large-scale system organization that were previously difficult to attain. The current study is unique in that it used both high-angular resolution DWI and rs-fcMRI to explore structural and functional rich club organization and how it develops. In agreement with prior reports [5], [6] we were able to show that global network architecture as measured by fiber tractography indeed has a rich club organization. We then demonstrated that this type of organization also applies to the functional brain as measured by correlated spontaneous activity, at least in typically developing populations. The rich club nodes for both data types were comprised of bilateral superior frontal and parietal cortex, together with anterior and posterior cingulate cortex, and the insula, comprising a number of previously identified brain systems [9]–[11], [37] including the default mode, fronto-parietal, cingulo-opercular, visual, and somatosensory systems. Importantly, the structural rich club showed a highly integrated region set, while the functional rich club was much more segregated into the known functional systems – suggesting that *while at rest* functional rich club nodes are less involved in integrating information across systems. We concluded the investigation with a brief examination of rich club organization in a childhood sample, demonstrating structural stability with key functional differences across age (i.e. for some nodes the “rich get richer,” for others they “get rich”).

### Rich Club Organization in both Structural and Functional Connectomes

With regard to modalities, the general architecture of the rich club was quite similar for both structural and functional data in adults. Both data types identified rich club nodes comprising bilateral regions of the midline frontal, midline posterior, and insula cortex, although the exact positioning of the nodes differed slightly (see Figures 3a, 4a, and 5). These findings are in large agreement with other studies, which have identified correspondence of functional and structural connectivity in samples of healthy adults [16], [17], [38]–[40]. With that said, structural and functional connectivity also diverge in important ways. For instance, functional connectivity can be indirect, comprising both monosynaptic and polysynaptic connections [41]. Some of the overlap we observe might be related to the matching of connection density across both methods, which we performed in order to maximize comparability. This matching required thresholding the functional network and choosing only the strongest connections. It might be expected that monosynaptic connections underly the strongest functional links, which in turn might account for some of the similarity between the modalities.

Even so, some regions appear exclusively in the structural data (e.g. inferior temporal/fusiform cortex in the structural), while others exclusively in the functional data (e.g. lateral inferior parietal). Additionally, Figures 3 and 4 reveal that while the spatial patterns of the rich club for both structural and functional data cut across several systems, there were apparent differences in the patterns of cross-modular linking. In the structural data, rich club nodes link to other rich club nodes outside their own community or system (i.e. they don’t simply connect to their own community). On the other hand, the functional rich club has relatively strong segregation between systems, although there are some connections between the somatomotor and cingulo-opercular systems. The disparity in terms of structural integration vs. functional segregation was observed for children as well (data not shown). This phenomenon might be related to previous conjectures regarding the role of intrinsic correlated brain activity in maintaining network or system relationships [8], [25], [42]. Indeed, homeostasis in neural systems is central to proper functioning and has been a topic of inquiry for decades. Importantly, a as noted by Turrigiano and Nelson [43], along with various “housekeeping” mechanisms aimed at maintaining temperatures, electrolytes, pH, etc., neural activity itself is important for homeostatic regulation. They argue that without stabilizing, or homeostatic, mechanisms, such as spontaneous activity, selective changes in synaptic weights in the form of hebbian rules would drive evoked neural activity toward “runaway excitation or quiescence.” It might be reasoned that the cortical networks currently being described in the fcMRI literature during the resting condition, are reflective of similar or related homeostatic phenomena. With that said, undoubtedly the cortical work conducted within any given network during task conditions needs to be integrated for proper brain function. Along these lines several reports have proposed that phase synchronization via intrinsic activity of specific neural assemblies or networks is important for coordinating segregated and distributed neural processes. For example, Varela et al. [44] point out that terms such as bottom-up and top-down are only heuristics “for what is in reality a large-scale network that integrates both incoming and endogenous activity.” They continue, “it is precisely at this level where phase synchronization is crucial as a mechanism for large-scale integration.” With this in mind, the rich club nodes and the integration across systems of the structural networks potentially highlights the “highways” at which functional integration of this type might occur during specified task demands; however, because participants are “at rest” (not performing an explicit task here), the synchronization across these highways may not be observed. Such a view is consistent with recent work highlighting the dynamic task-dependent reorganization of multiple large-scale cognitive networks [45], [46]. Additional work across various task conditions will be able to further evaluate the notion of cross-systems integration in task contexts compared to rest (although see Fair et al. [47] highlighting difficulties toward this end).

Interestingly, our findings regarding low integration of functional rich nodes during rest are largely corroborated by two very recent studies. Using independent components analysis to define functionally rich nodes, Yu et al [48] reported relatively low overall resting functional connectivity between these nodes. Complimentary to these findings, Collin et al [22] showed that nodes participating in the structural rich club have low intrahemispheric functional connectivity relative to non-rich-club nodes. These studies demonstrate that rich clubs defined across modalities tend to have low functional integration.

On a related note, recent resting-state studies highlight the importance of identifying nodes that participate in multiple functional networks [49], [50]. These reports advocate for the investigation of so-called “transmodal” regions as brain hubs, rather than nodes of high degree. Nodes identified by these studies are also distributed, but differ somewhat from reports examining nodes of high degree. Additional work across species provides multiple lines of evidence that nodes within the brain’s structural rich clubs coincide with areas of overlapping functional systems [51], [52]. In line with our resuts, these reports support the notion that structural rich clubs play a crucial role in the integration of functionally segregated domains. Again, future work such as lesion studies or functional activation paradigms can directly test predictions about whether these nodes contribute to cross-systems integration during task conditions.

### Structural Rich Club Organization is Present in Childhood

In comparing rich club organization across age, we find little evidence of meaningful change in the structural networks. There appears to be robust rich club organization in both children and adults, and the spatial distribution of rich club regions bore obvious similarities across the groups (see Figure 6). With the exception of one degree-point (Figure S2), significant differences between child and adult groups were not present, although there appears to be a trend towards greater rich club organization in children. This result is not readily interpretable. While others have identified protracted microstructural white matter changes that occur over this age range [18], [53], [54], the data here suggests that despite the reduced myelination and FA, there is enough sensitivity of the tractography algorithms to identify the major fiber bundles in both populations. However, it is possible that alternative tractography approaches (i.e. probabilistic, see below) may be more sensitive to these changes. While we hesitate to make definitive conclusions about null findings, these findings suggest that the general architecture of the structural core identified by Heuvel and Sporns, and corroborated by this study, is likely in place by late childhood and relatively stable into adulthood.

### Functional Rich Club Organization Increases across Development

In contrast to the structural results, we do find evidence that the rich club organization of functional networks increases across age (qualified by noting a small gender difference in our adult and child sample – see Materials and Methods). Our results showed a significant difference between the rich club coefficients of adults and children across a broad range of k. These changes can be described as some nodes becoming “richer” (e.g. insula cortex) in adulthood relative to childhood, and some nodes absent in childhood that “get rich” (e.g. supramarginal cortex, dorsal aspect of superior parietal cortex) by adulthood (Figure 7). However, these findings do not directly demonstrate which connections contribute to these changes. Furthermore, since the rich club coefficient is a normalized metric, the interpretation of a difference in values is complex. To investigate brain regions which might contribute to these differences, we indepedently identified a bilateral cluster of connections between superior parietal and insula cortex that were significantly stronger in adults. Nearly all nodes within this cluster participate in the adult functional rich club. Furthermore, this cluster is predominantly comprised of the nodes that became “richer” (insula) and those that got “rich” (supramarginal, superior parietal cortex), suggesting that over development, the most prominent functional changes that we can identify are those that enhance rich club organization. Lastly, this cluster includes some of the few connections which serve to integrate between the somatosensory and cingulo-opercular systems within the functional rich club (Figure 4), which is otherwise highly segregated. Taken together, these results suggest that this developmental period involves the strengthening of a particular set of connections which serve to integrate information across key hub nodes, and potentially across distinct functional systems.

Overall, these results indicate that, spanning this developmental period, the most prominent structural tracts are in place while functional relationships evolve. This notion agrees with previous work regarding developing functional brain networks. Hwang et al. have identified a particular set of connections to high-degree nodes, which they term “spoke” connections, that strengthens across adolescence [19]. Similarly, combined diffusion and resting-state imaging work has demonstrated that the brain’s major cortical white-matter tracts give rise to functional connections which strengthen across this age range [55], and that functional coupling between key network nodes tends to be greater in adults. Together with results presented here, this work suggests that functional remodeling of hub connections occurs over this developmental time period, is supported by underlying structural pathways, and may be associated with increased demands for flexible and complex cognition in adulthood.This is somewhat supported by recent work linking structural and functional rich club alterations with Schizophrenia [48], [56]. Future studies could further test these predictions by comparing rich club dynamics during the task and rest conditions.

### Limitations

There are several methodological limitations to this study. One consideration is that rich club quantification was not performed at the level of the single subject. The analyses in this study, and commonly in related studies, were conducted on group-averaged matrices, which were thoroughly filtered according to steps evaluated in prior work. Establishing appropriate and thoroughly evaluated methods of obtaining accurate and reliable networks (and therefore rich clubs) within single subjects remains a critical challenge for the field. This is particularly a concern with regard to structural networks, where noise within DWI scans can and do lead to estimation of spurious tracts. While this concern is beyond the scope of this particular study, establishing optimal methods to assess intersubject variability of these measures will represent a crucial advancement for the field.

Similarly, the small sample size of this study presents a limitation. To validate and explore these findings as potential markers of development, future studies will require the inclusion of larger subject pools at multiple timepoints. This will allow for tighter control of potential confounds (e.g. gender, brain size, head motion), more precise estimates of node-network interactions, and greater insights regarding longitudinal trajectories.

Lastly, future research should assess different approaches to construct connectivity matrices. For instance, probabilistic tractography allows for the construction of full structural connectivity matrices, which may facilitate comparison with functional data (although this may present complexitiy in the interpretation of low-to-medium-probability connections). Likewise, region selection is another critical decision point that may affect connectivity analyses. While this study looked exclusively at corticocortical networks, subcortical and cerebellar regions will undoubtedly contribute to integration of corticocortical networks at both the structural and functional level. It also is likely that a functionally derived parcellation (see [10], [11]) would reveal unique information relative to the anatomically defined parcellation used here, although see [1] regarding difficulties posed by parcellations with unequal region sizes.

### Conclusions

The results presented here suggest two broad hypotheses to be validated and explored. First, it appears rich club organization of structural connections is in place by late childhood and stable across the ensuing period of development until early-to-mid adulthood. Second, we showed that rich club organization exists in functional brain networks during childhood, and strengthens and modifies in important ways across this same period. Testing these findings across different ages, including during adolescence, will help to better chart the trajectory of rich club organization throughout development, and to establish its stability across and within individuals. It will also be critical to determine how the identified network phenomenon relates to various behavioral and cognitive measures, and whether deviations from this trajectory are predictive of specific psychiatric disorders. The findings presented here provide a foundation for examining structural and functional rich club phenomena in multiple contexts.

## Supporting Information

Figure S1 **Functional rich club curves in adults and children using HQS normalization.** Unweighted rich club coefficients relative to random are shown for children (pink) and adults (red). Normalization was performed using the Hirschberger-Qi-Steuer (H-Q-S) algorithm, as opposed to the Maslov-Sneppen rewiring used for all figures in the main text. Asterisks denote significantly greater than random values (P<.05, permutation testing). Curves demonstrate significant values across a broad range in both groups, but greater values and a broader range in adults.
(TIFF)

Figure S2 **Adults versus children comparisons of rich club coefficients using group-randomizations.** Rich club curves reflect the same data as that presented in the main text. In order to compute significance values here, a null distribution of differences was obtained by randomizing group assignments as described in the methods and materials. Normalized rich club coefficients for structural data (left) and functional data (right). Color-coding shows weighted (adults = red, solid; children = pink, solid), and unweighted (adults = brown, dashed; children = tan, dashed) networks. Significant differences are indicated with an asterisk at the top of the graph for weighted networks, and at the bottom for unweighted.
(TIFF)

Figure S3 **Group differences in functional rich club coefficients persist across distinct connection densities.** **(a)** Normalized rich club coefficients for functional data are shown for unweighted networks (adults = red, children = pink) at multiple connection densities (4% = top left, 6% = top right, 8% = bottom left, 10% = bottom right). Significant differences, indicated with asterisks, are observed (adults>children) across a wide range of k at on each graph.
(TIFF)

Table S1 **List of regions used for analysis.**
(DOCX)

Table S2 **Group differences in rich club coefficients are insensitive to removal of BOLD scan frames.** BOLD frame removal was carried out in adults using two distinct methods: 1) random removal and 2) removal of latter scan portion. Frames were removed from each subject until the amount of remaining scan time did not exceed a particular threshold (15 min, 10 min, or 5 min). Tabulated values represent degree thresholds (K) at which significant differences in rich club coefficients (adults>children) were observed. Opposite differences were not observed. These values are compared to differences displayed in figure 7 (K = 5, 7–17, 19, 20), which are closely matched here.
(DOCX)

## References

[1] Bullmore E, Sporns O (2009) Complex brain networks: graph theoretical analysis of structural and functional systems. Nature reviews Neuroscience 10: 186–198. 19190637 10.1038/nrn2575

[2] Rubinov M, Sporns O (2010) Complex network measures of brain connectivity: uses and interpretations. Neuroimage 52: 1059–1069. 19819337 10.1016/j.neuroimage.2009.10.003

[3] Bassett DS, Bullmore ET (2009) Human brain networks in health and disease. Curr Opin Neurol 22: 340–347. 19494774 10.1097/WCO.0b013e32832d93dd PMC2902726

[4] Colizza V, Flammini A, Serrano MA, Vespignani A (2006) Detecting rich-club ordering in complex networks. Nature physics 2: 110–115.

[5] van den Heuvel MP, Sporns O (2011) Rich-club organization of the human connectome. J Neurosci 31: 15775–15786. 22049421 10.1523/JNEUROSCI.3539-11.2011 PMC6623027

[6] van den Heuvel MP, Kahn RS, Goni J, Sporns O (2012) High-cost, high-capacity backbone for global brain communication. Proc Natl Acad Sci U S A 109: 11372–11377. 22711833 10.1073/pnas.1203593109 PMC3396547

[7] Biswal B, Yetkin FZ, Haughton VM, Hyde JS (1995) Functional connectivity in the motor cortex of resting human brain using echo-planar MRI. Magn Reson Med 34: 537–541. 8524021 10.1002/mrm.1910340409

[8] Fox MD, Raichle ME (2007) Spontaneous fluctuations in brain activity observed with functional magnetic resonance imaging. Nat Rev Neurosci 8: 700–711. 17704812 10.1038/nrn2201

[9] Fair DA, Dosenbach NUF, Church JA, Cohen AL, Brahmbhatt S, et al (2007) Development of distinct control networks through segregation and integration. Proc Natl Acad Sci U S A 104: 13507–13512. 17679691 10.1073/pnas.0705843104 PMC1940033

[10] Power JD, Cohen AL, Nelson SM, Wig GS, Barnes KA, et al (2011) Functional network organization of the human brain. Neuron 72: 665–678. 22099467 10.1016/j.neuron.2011.09.006 PMC3222858

[11] Yeo BT, Krienen FM, Sepulcre J, Sabuncu MR, Lashkari D, et al (2011) The organization of the human cerebral cortex estimated by intrinsic functional connectivity. Journal of Neurophysiology 106: 1125–1165. 21653723 10.1152/jn.00338.2011 PMC3174820

[12] Stevens AA, Tappon SC, Garg A, Fair DA (2012) Functional brain network modularity captures inter- and intra-individual variation in working memory capacity. PLoS One 7: e30468. 22276205 10.1371/journal.pone.0030468 PMC3262818

[13] Cherkassky VL, Kana RK, Keller TA, Just MA (2006) Functional connectivity in a baseline resting-state network in autism. Neuroreport 17: 1687–1690. 17047454 10.1097/01.wnr.0000239956.45448.4c

[14] Fair DA, Posner J, Nagel BJ, Bathula D, Dias TG, et al (2010) Atypical default network connectivity in youth with attention-deficit/hyperactivity disorder. Biological Psychiatry 68: 1084–1091. 20728873 10.1016/j.biopsych.2010.07.003 PMC2997893

[15] Fornito A, Zalesky A, Pantelis C, Bullmore ET (2012) Schizophrenia, neuroimaging and connectomics. Neuroimage 62: 2296–2314. 22387165 10.1016/j.neuroimage.2011.12.090

[16] Hagmann P, Cammoun L, Gigandet X, Meuli R, Honey CJ, et al (2008) Mapping the structural core of human cerebral cortex. PLoS Biol 6: e159. 18597554 10.1371/journal.pbio.0060159 PMC2443193

[17] Honey CJ, Thivierge JP, Sporns O (2010) Can structure predict function in the human brain? Neuroimage 52: 766–776. 20116438 10.1016/j.neuroimage.2010.01.071

[18] Hagmann P, Sporns O, Madan N, Cammoun L, Pienaar R, et al (2010) White matter maturation reshapes structural connectivity in the late developing human brain. Proceedings of National Academy of Sciences, USA 107: 19067–19072. 10.1073/pnas.1009073107 PMC2973853 20956328

[19] Hwang K, Hallquist MN, Luna B (2013) The development of hub architecture in the human functional brain network. Cereb Cortex 23: 2380–2393. 22875861 10.1093/cercor/bhs227 PMC3767958

[20] Zalesky A, Fornito A, Harding IH, Cocchi L, Yucel M, et al (2010) Whole-brain anatomical networks: does the choice of nodes matter? Neuroimage 50: 970–983. 20035887 10.1016/j.neuroimage.2009.12.027

[21] Hagmann P, Grant PE, Fair DA (2012) MR connectomics: a conceptual framework for studying the developing brain. Front Syst Neurosci 6: 43. 22707934 10.3389/fnsys.2012.00043 PMC3374479

[22] Collin G, Sporns O, Mandl RC, van den Heuvel MP (2013) Structural and Functional Aspects Relating to Cost and Benefit of Rich Club Organization in the Human Cerebral Cortex. Cereb Cortex. 10.1093/cercor/bht064 PMC4128699 23551922

[23] Tuch DS, Reese TG, Wiegell MR, Wedeen VJ (2003) Diffusion MRI of complex neural architecture. Neuron 40: 885–895. 14659088 10.1016/s0896-6273(03)00758-x

[24] Fair DA, Nigg JT, Iyer S, Bathula D, Mills KL, et al (2012) Distinct neural signatures detected for ADHD subtypes after controlling for micro-movements in resting state functional connectivity MRI data. Front Syst Neurosci 6: 80. 23382713 10.3389/fnsys.2012.00080 PMC3563110

[25] Fox MD, Snyder AZ, Vincent JL, Corbetta M, Van Essen DC, et al (2005) The human brain is intrinsically organized into dynamic, anticorrelated functional networks. Proc Natl Acad Sci U S A 102: 9673–9678. 15976020 10.1073/pnas.0504136102 PMC1157105

[26] Hallquist MN, Hwang K, Luna B (2013) The nuisance of nuisance regression: spectral misspecification in a common approach to resting-state fMRI preprocessing reintroduces noise and obscures functional connectivity. Neuroimage 82: 208–225. 23747457 10.1016/j.neuroimage.2013.05.116 PMC3759585

[27] Power JD, Barnes KA, Snyder AZ, Schlaggar BL, Petersen SE (2012) Spurious but systematic correlations in functional connectivity MRI networks arise from subject motion. Neuroimage 59: 2142–2154. 22019881 10.1016/j.neuroimage.2011.10.018 PMC3254728

[28] Newman ME (2006) Modularity and community structure in networks. Proc Natl Acad Sci U S A 103: 8577–8582. 16723398 10.1073/pnas.0601602103 PMC1482622

[29] Zhou S, Mondragón RJ (2004) The rich-club phenomenon in the Internet topology. Communications Letters, IEEE 8: 180–182.

[30] Opsahl T, Colizza V, Panzarasa P, Ramasco JJ (2008) Prominence and control: the weighted rich-club effect. Phys Rev Lett 101: 168702. 18999722 10.1103/PhysRevLett.101.168702

[31] Zalesky A, Fornito A, Bullmore E (2012) On the use of correlation as a measure of network connectivity. Neuroimage 60: 2096–2106. 22343126 10.1016/j.neuroimage.2012.02.001

[32] van den Heuvel MP, Mandl RC, Stam CJ, Kahn RS, Hulshoff Pol HE (2010) Aberrant frontal and temporal complex network structure in schizophrenia: a graph theoretical analysis. J Neurosci 30: 15915–15926. 21106830 10.1523/JNEUROSCI.2874-10.2010 PMC6633761

[33] Zalesky A, Fornito A, Bullmore ET (2010) Network-based statistic: identifying differences in brain networks. Neuroimage 53: 1197–1207. 20600983 10.1016/j.neuroimage.2010.06.041

[34] Fornito A, Yoon J, Zalesky A, Bullmore ET, Carter CS (2011) General and specific functional connectivity disturbances in first-episode schizophrenia during cognitive control performance. Biol Psychiatry 70: 64–72. 21514570 10.1016/j.biopsych.2011.02.019 PMC4015465

[35] Cocchi L, Bramati IE, Zalesky A, Furukawa E, Fontenelle LF, et al (2012) Altered functional brain connectivity in a non-clinical sample of young adults with attention-deficit/hyperactivity disorder. J Neurosci 32: 17753–17761. 23223295 10.1523/JNEUROSCI.3272-12.2012 PMC6621678

[36] Zalesky A, Fornito A, Seal ML, Cocchi L, Westin CF, et al (2011) Disrupted axonal fiber connectivity in schizophrenia. Biol Psychiatry 69: 80–89. 21035793 10.1016/j.biopsych.2010.08.022 PMC4881385

[37] Dosenbach NU, Fair DA, Cohen AL, Schlaggar BL, Petersen SE (2008) A dual-networks architecture of top-down control. Trends in Cognitive Sciences 12: 99–105. 18262825 10.1016/j.tics.2008.01.001 PMC3632449

[38] Greicius MD, Supekar K, Menon V, Dougherty RF (2009) Resting-state functional connectivity reflects structural connectivity in the default mode network. Cerebral Cortex 19: 72–78. 18403396 10.1093/cercor/bhn059 PMC2605172

[39] Honey CJ, Sporns O, Cammoun L, Gigandet X, Thiran JP, et al (2009) Predicting human resting-state functional connectivity from structural connectivity. Proc Natl Acad Sci U S A 106: 2035–2040. 19188601 10.1073/pnas.0811168106 PMC2634800

[40] van den Heuvel MP, Mandl RC, Kahn RS, Hulshoff Pol HE (2009) Functionally linked resting-state networks reflect the underlying structural connectivity architecture of the human brain. Hum Brain Mapp 30: 3127–3141. 19235882 10.1002/hbm.20737 PMC6870902

[41] Vincent JL, Patel GH, Fox MD, Snyder AZ, Baker JT, et al (2007) Intrinsic functional architecture in the anesthetized monkey brain. Nature 447: 46–47. 17476267 10.1038/nature05758

[42] Raichle ME (2009) A paradigm shift in functional brain imaging. Journal of Neuroscience 29: 12729–12734. 19828783 10.1523/JNEUROSCI.4366-09.2009 PMC6665302

[43] Turrigiano GG, Nelson SB (2004) Homeostatic plasticity in the developing nervous system. Nat Rev Neurosci 5: 97–107. 14735113 10.1038/nrn1327

[44] Varela F, Lachaux J-P, Rodriguez E, Martinerie J (2001) The brainweb: phase synchronization and large-scale integration. Nature Reviews Neuroscience 2: 229–239. 11283746 10.1038/35067550

[45] Leech R, Sharp DJ (2013) The role of the posterior cingulate cortex in cognition and disease. Brain. 10.1093/brain/awt162 PMC3891440 23869106

[46] Cocchi L, Zalesky A, Fornito A, Mattingley JB (2013) Dynamic cooperation and competition between brain systems during cognitive control. Trends Cogn Sci 17: 493–501. 24021711 10.1016/j.tics.2013.08.006

[47] Fair DA, Schlaggar BL, Cohen AL, Miezin FM, Dosenbach NU, et al (2007) A method for using blocked and event-related fMRI data to study “resting state” functional connectivity. Neuroimage 35: 396–405. 17239622 10.1016/j.neuroimage.2006.11.051 PMC2563954

[48] Yu Q, Sui J, Liu J, Plis SM, Kiehl KA, et al (2013) Disrupted correlation between low frequency power and connectivity strength of resting state brain networks in schizophrenia. Schizophr Res 143: 165–171. 23182443 10.1016/j.schres.2012.11.001 PMC3540119

[49] Braga RM, Sharp DJ, Leeson C, Wise RJ, Leech R (2013) Echoes of the brain within default mode, association, and heteromodal cortices. J Neurosci 33: 14031–14039. 23986239 10.1523/JNEUROSCI.0570-13.2013 PMC3810536

[50] Power JD, Schlaggar BL, Lessov-Schlaggar CN, Petersen SE (2013) Evidence for hubs in human functional brain networks. Neuron 79: 798–813. 23972601 10.1016/j.neuron.2013.07.035 PMC3838673

[51] van den Heuvel MP, Sporns O (2013) An anatomical substrate for integration among functional networks in human cortex. J Neurosci 33: 14489–14500. 24005300 10.1523/JNEUROSCI.2128-13.2013 PMC6618386

[52] de Reus MA, van den Heuvel MP (2013) Rich club organization and intermodule communication in the cat connectome. J Neurosci 33: 12929–12939. 23926249 10.1523/JNEUROSCI.1448-13.2013 PMC6619725

[53] Lenroot RK, Gogtay N, Greenstein DK, Wells EM, Wallace GL, et al (2007) Sexual dimorphism of brain developmental trajectories during childhood and adolescence. Neuroimage 36: 1065–1073. 17513132 10.1016/j.neuroimage.2007.03.053 PMC2040300

[54] Asato MR, Terwilliger R, Woo J, Luna B (2010) White Matter Development in Adolescence: A DTI Study. Cerebral Cortex. 10.1093/cercor/bhp282 PMC2923214 20051363

[55] Uddin LQ, Supekar KS, Ryali S, Menon V (2011) Dynamic reconfiguration of structural and functional connectivity across core neurocognitive brain networks with development. J Neurosci 31: 18578–18589. 22171056 10.1523/JNEUROSCI.4465-11.2011 PMC3641286

[56] van den Heuvel MP, Sporns O, Collin G, Scheewe T, Mandl RC, et al (2013) Abnormal rich club organization and functional brain dynamics in schizophrenia. JAMA Psychiatry 70: 783–792. 23739835 10.1001/jamapsychiatry.2013.1328

