# Supplementary figures and images for: Structural and Functional Rich Club Organization of the Brain in Children and Adults
Source: PLoS One. 2014 Feb 5;9(2):e88297. doi: 10.1371/journal.pone.0088297 (PMC3915050; doi:10.1371/journal.pone.0088297)

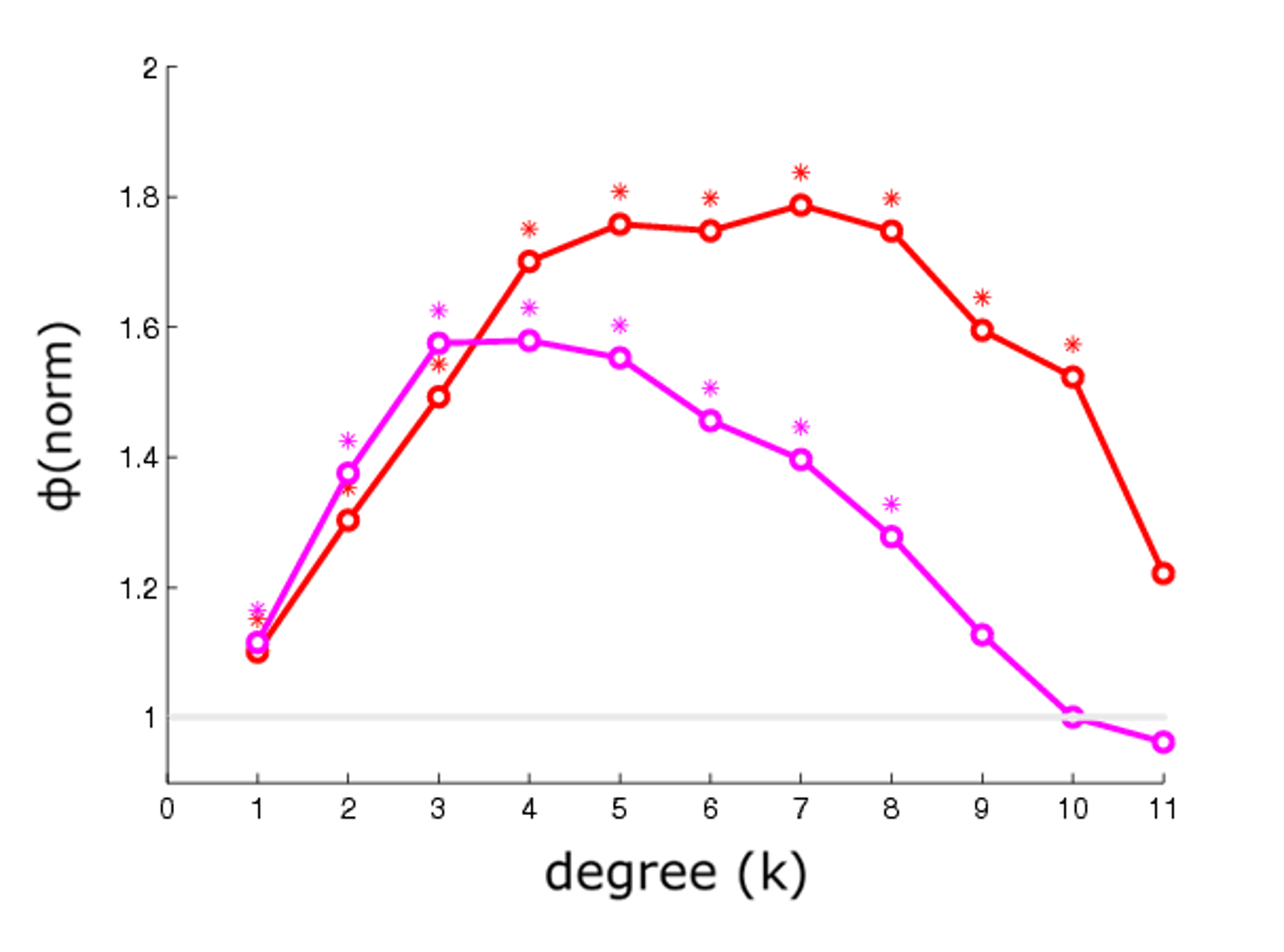

Supplement: Figure S1 — Functional rich club curves in adults and children using HQS normalization. Unweighted rich club coefficients relative to random are shown for children (pink) and adults (red). Normalization was performed using the Hirschberger-Qi-Steuer (H-Q-S) algorithm, as opposed to the Maslov-Sneppen rewiring used for all figures in the main text. Asterisks denote significantly greater than random values (P<.05, permutation testing). Curves demonstrate significant values across a broad range in both groups, but greater values and a broader range in adults. (TIFF) [file pone.0088297.s001.tiff]

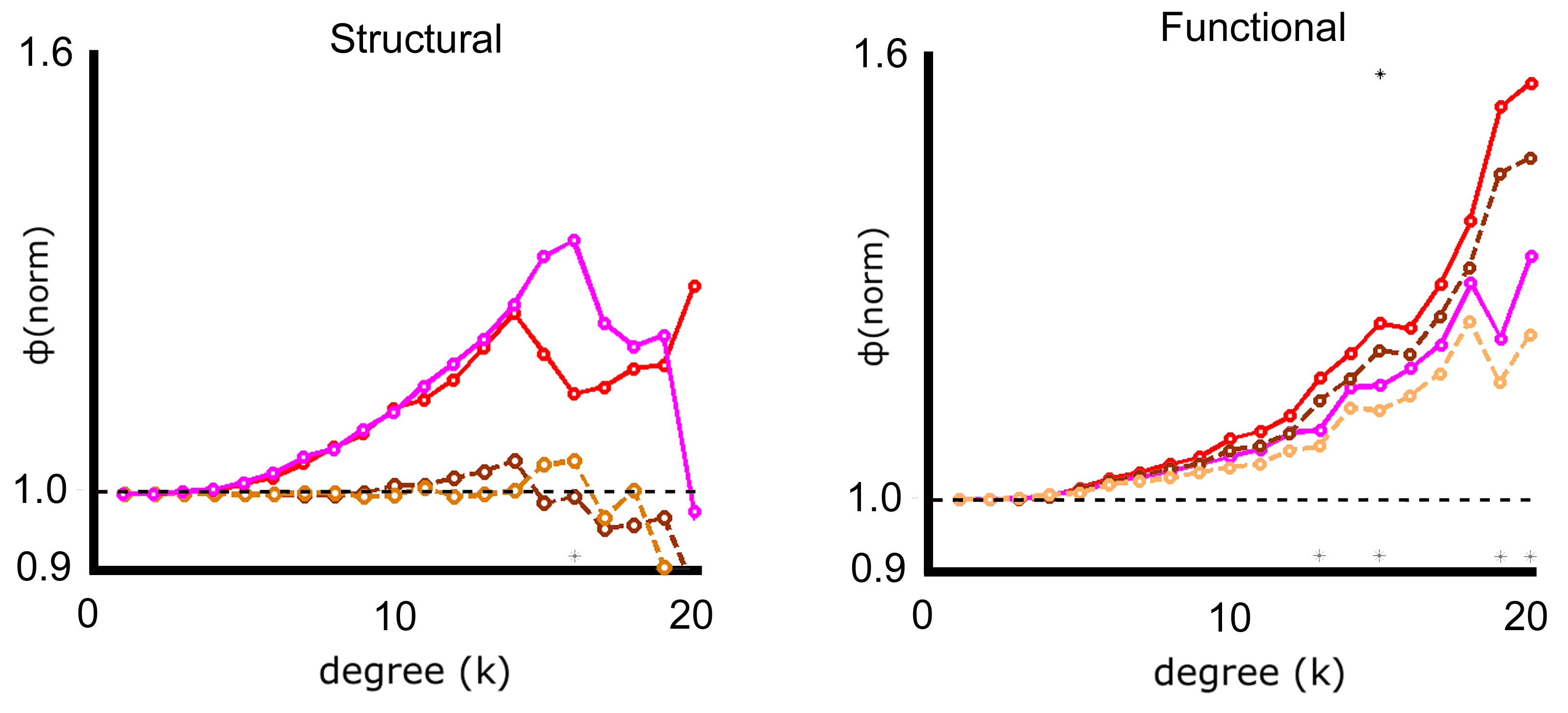

Supplement: Figure S2 — Adults versus children comparisons of rich club coefficients using group-randomizations. Rich club curves reflect the same data as that presented in the main text. In order to compute significance values here, a null distribution of differences was obtained by randomizing group assignments as described in the methods and materials. Normalized rich club coefficients for structural data (left) and functional data (right). Color-coding shows weighted (adults = red, solid; children = pink, solid), and unweighted (adults = brown, dashed; children = tan, dashed) networks. Significant differences are indicated with an asterisk at the top of the graph for weighted networks, and at the bottom for unweighted. (TIFF) [file pone.0088297.s002.tiff]

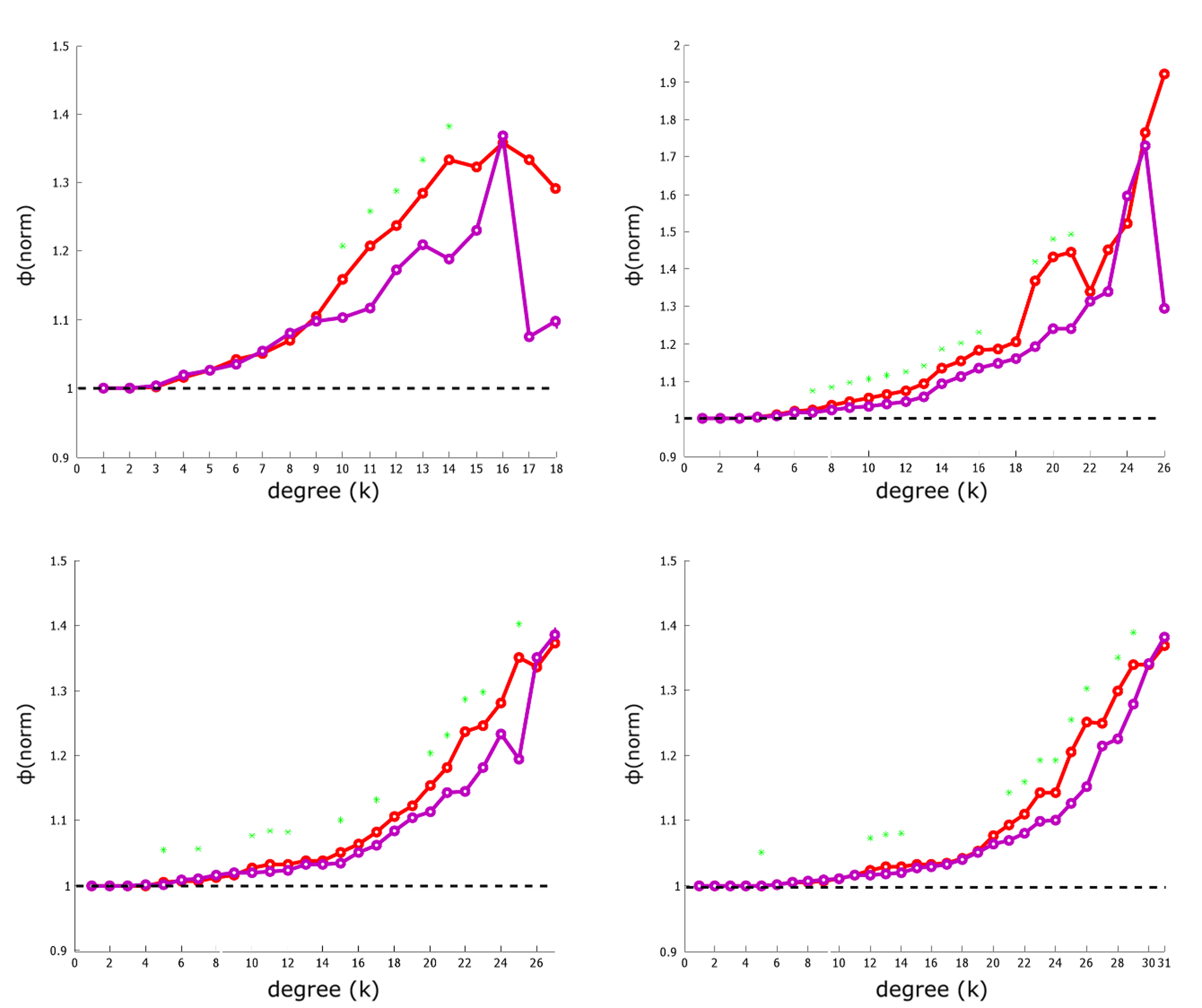

Supplement: Figure S3 — Group differences in functional rich club coefficients persist across distinct connection densities. (a) Normalized rich club coefficients for functional data are shown for unweighted networks (adults = red, children = pink) at multiple connection densities (4% = top left, 6% = top right, 8% = bottom left, 10% = bottom right). Significant differences, indicated with asterisks, are observed (adults>children) across a wide range of k at on each graph. (TIFF) [file pone.0088297.s003.tiff]
