# Supplementary material for: Structural and Functional Rich Club Organization of the Brain in Children and Adults
Source: PLoS One. 2014 Feb 5;9(2):e88297. doi: 10.1371/journal.pone.0088297 (PMC3915050; doi:10.1371/journal.pone.0088297)
Supplement: Table S1 — List of regions used for analysis. (DOCX) [file pone.0088297.s004.docx]

| **Left Hem** | **Right Hem** |
| --- | --- |
| lateralorbitofrontal_1 | lateralorbitofrontal_1 |
| lateralorbitofrontal_2 | lateralorbitofrontal_2 |
| lateralorbitofrontal_3 | lateralorbitofrontal_3 |
| lateralorbitofrontal_4 | lateralorbitofrontal_4 |
| parsorbitalis_1 | parsorbitalis_1 |
| frontalpole_1 | frontalpole_1 |
| medialorbitofrontal_1 | medialorbitofrontal_1 |
| medialorbitofrontal_2 | medialorbitofrontal_2 |
| parstriangularis_1 | medialorbitofrontal_3 |
| parsopercularis_1 | parstriangularis_1 |
| parsopercularis_2 | parstriangularis_2 |
| rostralmiddlefrontal_1 | parsopercularis_1 |
| rostralmiddlefrontal_2 | parsopercularis_2 |
| rostralmiddlefrontal_3 | rostralmiddlefrontal_1 |
| rostralmiddlefrontal_4 | rostralmiddlefrontal_2 |
| rostralmiddlefrontal_5 | rostralmiddlefrontal_3 |
| rostralmiddlefrontal_6 | rostralmiddlefrontal_4 |
| superiorfrontal_1 | rostralmiddlefrontal_5 |
| superiorfrontal_2 | rostralmiddlefrontal_6 |
| superiorfrontal_3 | superiorfrontal_1 |
| superiorfrontal_4 | superiorfrontal_2 |
| superiorfrontal_5 | superiorfrontal_3 |
| superiorfrontal_6 | superiorfrontal_4 |
| superiorfrontal_7 | superiorfrontal_5 |
| superiorfrontal_8 | superiorfrontal_6 |
| superiorfrontal_9 | superiorfrontal_7 |
| caudalmiddlefrontal_1 | superiorfrontal_8 |
| caudalmiddlefrontal_2 | caudalmiddlefrontal_1 |
| caudalmiddlefrontal_3 | caudalmiddlefrontal_2 |
| precentral_1 | caudalmiddlefrontal_3 |
| precentral_2 | precentral_1 |
| precentral_3 | precentral_2 |
| precentral_4 | precentral_3 |
| precentral_5 | precentral_4 |
| precentral_6 | precentral_5 |
| precentral_7 | precentral_6 |
| precentral_8 | paracentral_1 |
| paracentral_1 | paracentral_2 |
| paracentral_2 | paracentral_3 |
| rostralanteriorcingulate_1 | rostralanteriorcingulate_1 |
| caudalanteriorcingulate_1 | caudalanteriorcingulate_1 |
| posteriorcingulate_1 | posteriorcingulate_1 |
| posteriorcingulate_2 | posteriorcingulate_2 |
| isthmuscingulate_1 | isthmuscingulate_1 |
| postcentral_1 | postcentral_1 |
| postcentral_2 | postcentral_2 |
| postcentral_3 | postcentral_3 |
| postcentral_4 | postcentral_4 |
| postcentral_5 | postcentral_5 |
| postcentral_6 | supramarginal_1 |
| postcentral_7 | supramarginal_2 |
| supramarginal_1 | supramarginal_3 |
| supramarginal_2 | supramarginal_4 |
| supramarginal_3 | superiorparietal_1 |
| supramarginal_4 | superiorparietal_2 |
| supramarginal_5 | superiorparietal_3 |
| superiorparietal_1 | superiorparietal_4 |
| superiorparietal_2 | superiorparietal_5 |
| superiorparietal_3 | superiorparietal_6 |
| superiorparietal_4 | superiorparietal_7 |
| superiorparietal_5 | inferiorparietal_1 |
| superiorparietal_6 | inferiorparietal_2 |
| superiorparietal_7 | inferiorparietal_3 |
| inferiorparietal_1 | inferiorparietal_4 |
| inferiorparietal_2 | inferiorparietal_5 |
| inferiorparietal_3 | inferiorparietal_6 |
| inferiorparietal_4 | precuneus_1 |
| inferiorparietal_5 | precuneus_2 |
| precuneus_1 | precuneus_3 |
| precuneus_2 | precuneus_4 |
| precuneus_3 | precuneus_5 |
| precuneus_4 | cuneus_1 |
| precuneus_5 | cuneus_2 |
| cuneus_1 | pericalcarine_1 |
| pericalcarine_1 | pericalcarine_2 |
| lateraloccipital_1 | lateraloccipital_1 |
| lateraloccipital_2 | lateraloccipital_2 |
| lateraloccipital_3 | lateraloccipital_3 |
| lateraloccipital_4 | lateraloccipital_4 |
| lateraloccipital_5 | lateraloccipital_5 |
| lingual_1 | lingual_1 |
| lingual_2 | lingual_2 |
| lingual_3 | lingual_3 |
| lingual_4 | fusiform_1 |
| fusiform_1 | fusiform_2 |
| fusiform_2 | fusiform_3 |
| fusiform_3 | fusiform_4 |
| fusiform_4 | parahippocampal_1 |
| parahippocampal_1 | entorhinal_1 |
| entorhinal_1 | temporalpole_1 |
| temporalpole_1 | inferiortemporal_1 |
| inferiortemporal_1 | inferiortemporal_2 |
| inferiortemporal_2 | inferiortemporal_3 |
| inferiortemporal_3 | inferiortemporal_4 |
| inferiortemporal_4 | middletemporal_1 |
| middletemporal_1 | middletemporal_2 |
| middletemporal_2 | middletemporal_3 |
| middletemporal_3 | middletemporal_4 |
| middletemporal_4 | bankssts_1 |
| bankssts_1 | superiortemporal_1 |
| bankssts_2 | superiortemporal_2 |
| superiortemporal_1 | superiortemporal_3 |
| superiortemporal_2 | superiortemporal_4 |
| superiortemporal_3 | superiortemporal_5 |
| superiortemporal_4 | transversetemporal_1 |
| superiortemporal_5 | insula_1 |
| transversetemporal_1 | insula_2 |
| insula_1 | insula_3 |
| insula_2 |  |
| insula_3 |  |
| insula_4 |  |
