# Supplementary material for: Structural and Functional Rich Club Organization of the Brain in Children and Adults
Source: PLoS One. 2014 Feb 5;9(2):e88297. doi: 10.1371/journal.pone.0088297 (PMC3915050; doi:10.1371/journal.pone.0088297)
Supplement: Table S2 — Group differences in rich club coefficients are insensitive to removal of BOLD scan frames. BOLD frame removal was carried out in adults using two distinct methods: 1) random removal and 2) removal of latter scan portion. Frames were removed from each subject until the amount of remaining scan time did not exceed a particular threshold (15 min, 10 min, or 5 min). Tabulated values represent degree thresholds (K) at which significant differences in rich club coefficients (adults>children) were observed. Opposite differences were not observed. These values are compared to differences displayed in figure 7 (K = 5, 7–17, 19, 20), which are closely matched here. (DOCX) [file pone.0088297.s005.docx]

|  | **Frame removal method** | |
| --- | --- | --- |
| **Max time remaining** | **Randomized** | **Latter portion** |
| 15min | K=9-15, 19 | K=4, 7-20 |
| 10min | K=5,7-11,13,15-17,18 | K=5-18, 20 |
| 5min | K=4 ,5, 8-13, 15-17,19 | K=5, 7-19 |
